# Moon Rock Cannabis-Induced Psychosis and New-Onset Seizures in a 20-Year-Old Male

**DOI:** 10.7759/cureus.42752

**Published:** 2023-07-31

**Authors:** Martin Leczycki, Peter Zaki, Eduardo D Espiridion

**Affiliations:** 1 Psychiatry, Reading Hospital - Tower Health, West Reading, USA; 2 Neurosurgery, Drexel University College of Medicine, Philadelphia, USA; 3 Psychiatry, Drexel University College of Medicine, Philadelphia, USA

**Keywords:** tetrahydrocannabinol, tetrahydrocannabinol (thc), psychosis, seizure, cannabis, moon rocks

## Abstract

“Moon rock” cannabis is a type of new and highly potent preparation of cannabis, which is made of a strain of cannabis dipped in hash oil and sprinkled with kief crystals, effectively increasing the concentration of δ-9-tetrahydrocannabinol (THC), the main psychoactive compound in cannabis, well beyond what is naturally found in the cannabis plant. The use of increasingly potent forms of cannabis has far-reaching health implications, including psychiatric and neurologic effects, which are not yet fully understood. This case report summarizes existing knowledge of the association of cannabis use with psychosis and seizures and describes a novel case of “moon rock” cannabis-induced psychosis and new-onset seizures.

## Introduction

For centuries, plants from the genus Cannabis have been used for the effects of the psychoactive compounds they contain, of which the cannabinoid δ-9-tetrahydrocannabinol (THC) is the most prominent and well-studied. There are multiple isomers of THC, both naturally and synthetically formed, though what they have in common is their action on the G protein-coupled cannabinoid receptors, CB1 and CB2, found in the brain and throughout the body [1,2]. THC is a partial agonist at the CB1 receptor, which by its activation, causes inhibition of presynaptic neurotransmitter release and a resulting decrease in signal propagation in the dopaminergic, GABAergic, glutamatergic, serotonergic, norepinephrinergic, and cholinergic systems. Because of the involvement of these neurotransmitter systems, CB1 receptor activity can affect functions including cognition, memory, motor movements, and pain perception [2,3]. Cannabis use is an area of study that has seen increased attention in recent years, to better understand the endocannabinoid receptor system, ways in which it can be used therapeutically, and how it may be involved in neuro- and psychopathology. Compounds acting at cannabinoid receptors afford some medicinal properties, showing therapeutic potential as appetite stimulants, antiemetics, analgesics, and muscle relaxants [4,5]. Exposure to cannabis can also have undesirable physical, mental, and behavioral effects. Acute cannabis use is associated with impaired attention, psychomotor task ability, and memory, effects that may persist with long-term use [3]. It has also been found to have varying effects on mood, anxiety, and psychotic disorders. At an epidemiologic level, a dose-response relationship has been found between cannabis use and increased risk of psychosis [6,7]. While some forms of cannabis, especially those that predominantly contain the cannabinoid cannabidiol (CBD), have evidence to suggest efficacy as antiepileptic agents [3], forms with increased THC concentration, and likewise agonism on the CB1 receptor, have been reported to be associated with seizure activity. This has been seen particularly with synthetic forms of cannabis, which are known to have a higher THC concentration than naturally occurring cannabis [8]. THC concentration in dispensary-sold cannabis ranges from 17% to 28% but can be upwards of 50% in synthetic cannabis [9]. The form factor, or the way in which cannabis is prepared for use, may also increase the THC content. For example, one emerging variety of preparation called “moon rocks” consists of a potent strain of cannabis dipped in hash oil and sprinkled with kief crystals, effectively increasing the potency of the consumed product [10]. Since they contain a higher THC concentration, consumers may look to “moon rocks” to achieve a more intense "high" but by the same token, this preparation may induce undesired levels of intoxication. In this case report, we present a novel case of “moon rock” cannabis-induced psychosis and new-onset seizures.

## Case presentation

A 20-year-old man with no significant past medical history or psychiatric history presented to the hospital for evaluation of bizarre behavior and seizure-like activity at home. Per the patient’s family, the patient was smoking “moon rock” cannabis and became anxious and agitated, slit his wrist with a knife, and attempted to choke his brother. He then had a loss of consciousness with seizure-like activity described by his mother as shaking and stiffening of his extremities for several minutes, with his eyes rolling back and foam coming from his mouth. He was brought to the emergency department (ED) by emergency medical services (EMS), who administered 5 milligrams of midazolam intramuscularly. On arrival at the ED, his vital signs were normal, though he was lethargic and responsive only to painful stimuli. Shortly after, he awoke in a confused state and stated, “I am getting angry. They are hurting me. I am coming back stronger.” At this time, he reported hearing voices saying derogatory things to him. He was combative toward staff and pulled at his lines and leads. Soon after he awoke in the ED, he had a witnessed tonic-clonic seizure and was administered 2 milligrams of midazolam and 2 grams of levetiracetam intravenously.

Laboratory studies, which were taken on arrival at the ED, included complete blood count, complete metabolic panel, serum lactic acid, and alcohol levels, which were all within normal limits. The patient had an elevated serum creatine kinase level of 239 IU/L with a serum glucose level of 108 mg/dL. Urinalysis was unremarkable, though urine drug screen was positive for THC and benzodiazepines. Electrocardiogram showed a normal sinus rhythm. CT and MRI scans of the brain showed no intracranial abnormalities. A routine EEG performed after his seizure in the ED did not show any epileptiform activity (Table 1).

**Table 1:** Initial test results of the patient BUN: blood urea nitrogen; AST: aspartate aminotransferase; ALT: alanine aminotransferase; CK: creatine kinase; WBC: white blood cells; RBC: red blood cells; MCV: mean corpuscular volume; MCH: mean corpuscular hemoglobin; MCHC: mean corpuscular hemoglobin concentration; RDW: red cell distribution width; MPV: mean platelet volume; PCP: phencyclidine; THC: δ-9-tetrahydrocannabinol; ECG: electrocardiogram; CT: computed tomography; MRI: magnetic resonance imaging; EEG: electroencephalogram.

Test name	Result	Reference range
Sodium	140 mmol/L	136-145 mmol/L
Potassium	3.6 mmol/L	3.5-5.1 mmol/L
Chloride	105 mmol/L	98-107 mmol/L
CO2	28.4 mmol/L	21.0-31.0 mmol/L
Anion gap	7 mmol/L	5-12 mmol/L
BUN	7 mg/dL	7-25 mg/dL
Creatinine	0.78 mg/dL	0.60-1.30 mg/dL
Glucose	108 mg/dL	70-99 mg/dL
Calcium	9.2 mg/dL	8.6-10.3 mg/dL
Alkaline phosphatase	55 IU/L	34-104 IU/L
Albumin	4.3 g/dL	3.5-5.7 g/dL
Total protein	6.8 g/dL	6.4-8.9 g/dL
AST	22 IU/L	13-39 IU/L
ALT	9 IU/L	7-52 IU/L
Total bilirubin	0.4 mg/dL	0.3-1.0 mg/dL
Lactic acid	0.9 mmol/L	0.6-1.4 mmol/L
CK	239 IU/L	30-223 IU/L
Troponin	<0.03 ng/mL	<0.06 ng/mL
Magnesium	2.3 mg/dL	1.9-2.7 mg/dL
Phosphorus	4.1 mg/dL	2.5-5.0 mg/dL
Alcohol	<10 mg/dL	<10 mg/dL
WBC	5.9 x 10^3/µL	4.8-10.8 x 10^3/µL
RBC	4.72 x 10^6/µL	4.50-6.10 x 10^6/µL
Hemoglobin	14.1 g/dL	14.0-17.5 g/dL
Hematocrit	42%	39-53%
MCV	89 fL	80-99 fL
MCH	29.9 pg	27.0-34.0 pg
MCHC	33.6 g/dL	31.0-37.0 g/dL
RDW	12.90%	11.0-16.0%
Platelets	182 x 10^3/µL	130-400 x 10^3/µL
MPV	11.2 fL	8.0-13.0 fL
Neutrophil number	3.6 x 10^3/µL	2.00-8.00 x 10^3/µL
Neutrophil percent	61.3%	45-75%
Lymphocyte number	1.44 x 10^3/µL	0.70-5.20 x 10^3/µL
Lymphocyte percent	24.5%	19-46%
Monocyte number	0.59 x 10^3/µL	0.10-1.30 x 10^3/µL
Monocyte percent	10%	2-12%
Eosinophil number	0.2 x 10^3/µL	0.04-0.54 x 10^3/µL
Eosinophil percent	3.4%	0-4%
Basophil number	0.03 x 10^3/µL	0.00-0.21 x 10^3/µL
Basophil percent	0.5%	0-1.5%
Urine color	Yellow	-
Urine appearance	Clear	-
Urine pH	7	5.0-8.0
Urine specific gravity	1.015	1.005-1.028
Urine glucose	Negative	-
Urine ketones	Negative	-
Urine bilirubin	Negative	-
Urine blood	Negative	-
Urine protein	Negative	-
Urine urobilinogen	Normal	-
Urine leukocyte esterase	Negative	-
Urine nitrite	Negative	-
Urine opiate screen	Negative	-
Urine amphetamine screen	Negative	-
Urine methadone screen	Negative	-
Urine cocaine screen	Negative	-
Urine barbiturates screen	Negative	-
Urine benzodiazepine screen	Positive	-
Urine PCP screen	Negative	-
Urine THC screen	Positive	-
Urine oxycodone screen	Negative	-
Urine fentanyl screen	Negative	-
ECG 12-lead	Rate 66 bpm, QRS 72 ms, QTC 423 ms	-
CT brain	Normal	-
MRI brain	Normal	-
Routine EEG	No epileptiform activity identified	-

At the time of evaluation by neurology several hours later, the patient’s mental status had improved, and he was able to follow commands and have a conversation, though he was still mildly confused. His neurologic examination was non-focal. He denied a history of seizure-like episodes, but his mother reported she had a history of epilepsy. The patient also reported a past concussion with loss of consciousness from falling off his bike without a helmet at the age of 18 years, though denied any persistent concussive symptoms. He could not remember details immediately preceding his seizures at home or in the ED, though he reported confusion and muscle soreness after waking up. Neurology did not initiate scheduled anti-epileptic medication as it was deemed that the patient had a provoked seizure from “moon rock” use. While speaking with the neurologists, he reported recent self-injurious behavior, so psychiatry was consulted.

During the psychiatric evaluation, which occurred the day after his seizures, the patient was awake, alert, fully oriented, and no longer confused. He denied any past psychiatric history, evaluation, or treatment. He reported that he had been in his usual state of health and function recently with no medical conditions. Neither he nor his family had any past psychiatric history. He reported that prior to his hospitalization, he smoked several blunts containing “moon rocks” for the first time, though he had a history of frequent cannabis use since his mid-teens. He denied a history of cannabis withdrawal symptoms. Besides infrequent social consumption of alcohol, he denied any other recent substance use. He described the “moon rocks” as a highly potent strain of cannabis in powdered form that he added to his usual cannabis blunt. After smoking the “moon rocks,” he reported feeling extremely euphoric but otherwise reported an effect similar to that of his usual cannabis use and denied adverse effects. The next day, he smoked another blunt containing “moon rocks,” but this time reacted differently. He had an acute onset of anxiety, paranoia, got into an argument with his brother, and made a small cut on his wrist with a knife. He did not remember what his intent was when cutting himself but denied thoughts of harming himself currently and denied prior self-harm, suicidal ideation, or suicide attempt. After he cut himself, he did not have any memory of events until he awoke in the hospital, confused and agitated. He was not sure if he had auditory or visual hallucinations at any point but denied their presence at the time of the interview. His family corroborated that he had a seizure-like episode at home but had otherwise been in his usual state of health recently with no additional concerns or history provided. At the time of psychiatric evaluation, the patient did not have any acute psychiatric symptoms or needs, and he was discharged home once medically cleared.

The patient presented back to the ED the next day following another episode of seizure-like activity at home. His mother reported witnessing several minutes of seizure-like activity, appearing exactly like his previous episodes. EMS arrived to find him in a postictal state, administered midazolam intramuscularly, then took him to the ED. He and his mother denied any substance use since his discharge. Laboratory findings were unchanged from the prior, though he did not undergo EEG or imaging studies. Neurology evaluated him once again and started levetiracetam 500 milligrams twice daily.

Two weeks later, the patient presented back to the ED a third time following another episode of seizure-like activity witnessed by family at home. The patient and his mother both reported that he was compliant with his antiepileptic medication and denied any substance use. Exam and laboratory findings were mostly unchanged, now with a negative urine drug screen and a therapeutic serum level of levetiracetam. A routine EEG was performed and again did not show any epileptiform activity. Given his breakthrough seizure, the patient was discharged home on an increased dose of levetiracetam 1000 milligrams twice daily. As of two months after his most recent ED visit, the patient had no additional seizures, reporting that he was taking his medication and had refrained from substance use.

## Discussion

To summarize the case, the patient was a 20-year-old male with no past psychiatric history but frequent use of cannabis since his mid-teens, one prior concussion, and no history of seizure-like activity but with a mother with epilepsy, who had new-onset psychosis and seizures after smoking “moon rock” cannabis for the first time. His psychosis, characterized by heightened anxiety, paranoia, auditory hallucinations, and combative behavior, was brief and self-resolving. However, he had multiple generalized tonic-clonic seizures that were witnessed by family and hospital staff. Generalized tonic-clonic seizures, also known as grand mal seizures, are seizures characterized by a loss of consciousness followed by consecutive phases of tonic and clonic muscle contractions occurring bilaterally. They are the type of seizure most commonly associated with epilepsy. The patient's first seizure occurred one day after the initial use of “moon rock” cannabis, and he had additional seizures four days and 19 days after the initial seizure. It is unlikely that there was any other stimulus that could have provoked his seizures, as both the patient and family denied the use of any other substance or recent stressors, and his initial urine drug screen showed THC and benzodiazepines, the latter of which were administered en route to the hospital. He had no chronic medical conditions or evidence of an acute organic process from his laboratory studies. It is worth noting that the patient did have a history of concussions as well as a family history of epilepsy in his mother, which would have increased his risk of developing a seizure. This presents the possibility that the “moon rock” use was simply a marker or obvious incident to which the timing of events was being compared to. However, it would seem more likely that given the timeline of events reported by the patient, his family, and hospital staff, the seizures were in some way associated with “moon rock” use.

Characterizing the health effects of cannabis has been a subject of ongoing debate over the years. Studies have focused on the effects of cannabis on physical and mental health, though such studies are often limited by the illegal status of cannabis in most countries. However, multiple systematic reviews have suggested an association between cannabis use and the development of psychotic disorders, with studies showing that first-episode psychosis patients were more likely to be heavy, high-potency cannabis users, and more likely to have used cannabis in early adolescence [11]. Likewise, there is evidence to suggest an association between cannabis use and an increased incidence of suicidal ideation and suicide attempts, especially in heavy cannabis users and young adults [11]. Adolescence and early adulthood are the times when experimentation with substances of abuse, including cannabis, tends to begin. This is also a time characterized by a substantial development of both brain structure and function, including myelinization, synaptic pruning, and reduction of gray matter volume. The effects of cannabis exposure on the developing brain are thus thought to be significant and may have effects lasting into adulthood [9].

It is suspected that the three domains of cognition (attention, memory, and learning) are adversely affected by acute cannabis use, with evidence even suggesting that some effects can persist over time [3]. MRI and functional MRI studies in adults have shown differences in brain activity between chronic cannabis users and non-users, with chronic users demonstrating altered patterns of activity, particularly within the prefrontal cortex, while achieving similar task outcomes [12]. This would suggest that the effects of cannabis on cognition may involve a compensatory mechanism, recruiting additional areas of the brain and possibly altering neuronal and neurotransmitter activity in the long term. CB1 receptors are abundant in the brain and are particularly concentrated in areas involved in executive functioning, reward processing, and memory, such as the prefrontal cortex, anterior cingulate cortex, basal ganglia, hippocampus, and amygdala [12]. By its action on CB1 receptors, THC may affect the structure, connectivity, and function of these areas of the brain in which CB1 receptors are found. In fact, MRI studies have found cannabis users to have reduced volumes of the hippocampus and amygdala, areas of the brain known to be negatively impacted by the chronic use of addictive substances, a category in which cannabis is often included due to the psychoactive component of THC [9].

While the effects of cannabis use on psychological processes and pathology have received a fair amount of focus in the scientific literature, the association between cannabis use and seizures is by comparison poorly understood. Epidemiologic data have suggested that cannabis use can be a protective factor against new-onset seizures [13]. In fact, cannabis has been used for centuries as a treatment for epilepsy before the advent of modern antiepileptic medications, but the evidence supporting or refuting its efficacy remains largely anecdotal, with few scientific studies having been conducted [14]. Various studies in animal models have indicated both the anticonvulsant and proconvulsant effects of cannabis [5]. There have also been some reports of seizures associated with cannabis use, especially synthetic cannabis, which has become popular in recent years and typically has a THC content much greater than that found in naturally occurring cannabis [15,16]. By comparison, cannabidiol (CBD) is another cannabinoid compound naturally found in cannabis that has more evidence to suggest efficacy as an antiepileptic, having even received FDA approval for the treatment of Dravet syndrome and Lennox-Gastaut syndrome, two forms of pediatric epilepsy, as well as for seizures associated with tuberous sclerosis complex [3]. CBD is a negative allosteric modulator of the CB1 receptor, effectively opposing the psychoactive effects of THC, though the remaining molecular mechanisms by which CBD acts remain unclear [17]. While THC and CBD are found in varying proportions in the cannabis plant, the CBD content of “street” cannabis has been found to be extremely low in recent times, as cannabis cultivators have selected cannabis plants with increasing levels of THC over the years [2].

With so many different varieties of cannabis with different chemical compositions, it becomes increasingly difficult to characterize their effects as a whole. To date, there have been few large-scale, randomized, placebo-controlled trials that have tested the effects of cannabis and its constituent compounds on the incidence, treatment, or prevention of seizures. As such, this remains a topic needing further investigation due to cannabis use being a widespread and increasingly popular phenomenon, as well as the clinical relevance of seizures due to their neurobiological, cognitive, psychological, and social consequences.

## Conclusions

This case adds to the growing pool of evidence that cannabis use may be a risk factor for the development of seizures in addition to the comparatively more well-known effect of psychosis. Specifically, there is a focus on the association between THC concentration of cannabis and its adverse effects. “Moon rock” cannabis is a type of new and highly potent preparation of cannabis with a higher THC concentration than naturally found in the cannabis plant. The use of “moon rocks” and synthetic forms of cannabis presents troubling implications on neuro- and psychopathology. The effects of cannabis and THC on seizure pathology are poorly understood and remain a topic requiring further investigation to make clinical recommendations.
